# Notes and News

**Published:** 1900-07-14

**Authors:** 


### Notes and News.

The annual distribution of prizes to the students of St. Mary's Hospital Medical School took place on the 13th inst.

The National Association for Promoting the Welfare of the Feeble-minded have memorialised the Local Government Board that an inquiry may be instituted as to the far-reaching evils attending the want of proper supervision of feeble-minded paupers amongst the community.

Dr. Frazer, of Sydney, New South Wales, is now known to be the donor of the munificent gift of £5,000 towards the establishment of new pathological laboratories at Oxford. The gift remained for some time anonymous, but the donor has now allowed his name to be known.

A good deal of interest attended the last meeting of the Governors of Addenbrooke's Hospital, Cambridge. It was announced that a circular to members of the University, asking for further donations and subscriptions, had already borne good fruit. The medical staff are going to build themselves a room for research, relinquishing the apartment now used by them to be used as the matron's sitting-room. They also intend to bear the cost of additions to the mortuary. Mrs. Peart has undertaken the cost of adapting a colonnade on the first floor for the purpose of open-air treatment. Mr. Bennett has been re-elected as secretary, and Mr. Ellet has been elected as superintendent.

At the annual meeting of the Röntgen Society, which was held last week, Mr. Noble, the president, in detailing the various advances which had been made during the year, laid considerable stress on what had been done in regard to stereoscopic radioscopy—seeing objects in relief on the screen. They were now, he said, able to say that this was an accomplished fact; and, as showing its importance, he pointed out how much more easy it was to distinguish things with certainty when seen in relief than when they all appeared on a flat surface. More especially was this the case with objects showing but little contrast and ill-defined, such as early patches of tuberculosis in the lung, the diagnosis of which, he thought, would be enormously facilitated when visual stereoscopic radiography became general.

A cottage hospital has been opened at Milford-on-Sea.

A systematised campaign against malaria has been organised, and full instructions have been issued by the Bureau of Hygiene of Rome. These instructions give the procedure to be followed during each month. Prophylactic measures are recommended in detail, and rules for the treatment of malaria in its various stages are clearly set forth.

The Metropolitan Hospital Saturday Fund carry out a useful system of supplying ambulance boxes and litters on loan to workshops. In connection with these knowledge in first aid to the injured is disseminated as much as possible, and the means of immediate attention which the availability of appliances affords has proved most useful in a number of cases, even to the extent of actually saving life.

It is serious news that an outbreak of cholera has occurred amongst the native troops at Kohat, in India. Two hundred and seven cases occurred in a week amongst the Sepoys and camp followers. One regiment had fortunately left for China before the outbreak. The plague is still prevalent in many places. Hong Kong continues to record a considerable number of deaths. In Queensland the spread of the disease has not proved at all rapid, though the percentage of deaths is large.

In commemoration of 25 years of service as secretary of the Charity Organisation Society, Mr. C. S. Loch was last Monday presented with his portrait, painted by Mr. Sargent, R.A., together with the balance—over £1,000—of the fund which had been raised on the occasion. The presentation was made at Lambeth Palace by the Archbishop of Canterbury, who spoke in high terms of Mr. Loch's individual share in maintaining and advancing the principles of the society in face of much popular misunderstanding. The list of subscribers, numbering over 500, is representative of all classes and many nationalities.

The Hospital for Epilepsy and Paralysis has recently received from the executors of Mr. J. W. N. Bentley the munificent sum of £5,000, being part of the residue of the testator's estate placed at their disposal by him. This timely gift, when added to the new building fund, will raise the latter to a total of £12,600, but more than twice that amount will be required to erect, upon a site in the immediate neighbourhood of the hospital (Portland Terrace, Regent's Park), the negotiations for which are all but complete, a building adequate to the present and future needs of the hospital, and the requirements of modern hygiene and sanitation. The committee have selected as their architect Mr. Keith D. Young, F.R.I.B.A., a recognised expert in hospital construction. Towards the new building fund many liberal donations have been received, amongst others £250 from the Prince of Wales's Fund, and two of £500 each from Miss Harben and from Mr. D. Compton, a member of the committee. But many such are needed before the committee can feel justified in fully carrying out the new development of their work—forced upon them by the expiration of the lease of their premises.

